# Supplementary material for: Viral diversity and dynamics and CRISPR-Cas-mediated immunity in a robust alkaliphilic cyanobacterial consortium
Source: Microbiol Spectr. 2023 Oct 11;11(6):e02217-23. doi: 10.1128/spectrum.02217-23 (PMC10715143; doi:10.1128/spectrum.02217-23)
Supplement: Supplementary File — Supplementary figures and their captions and captions for supplementary tables. [file spectrum.02217-23-s0001.pdf]

Supplementary Figures

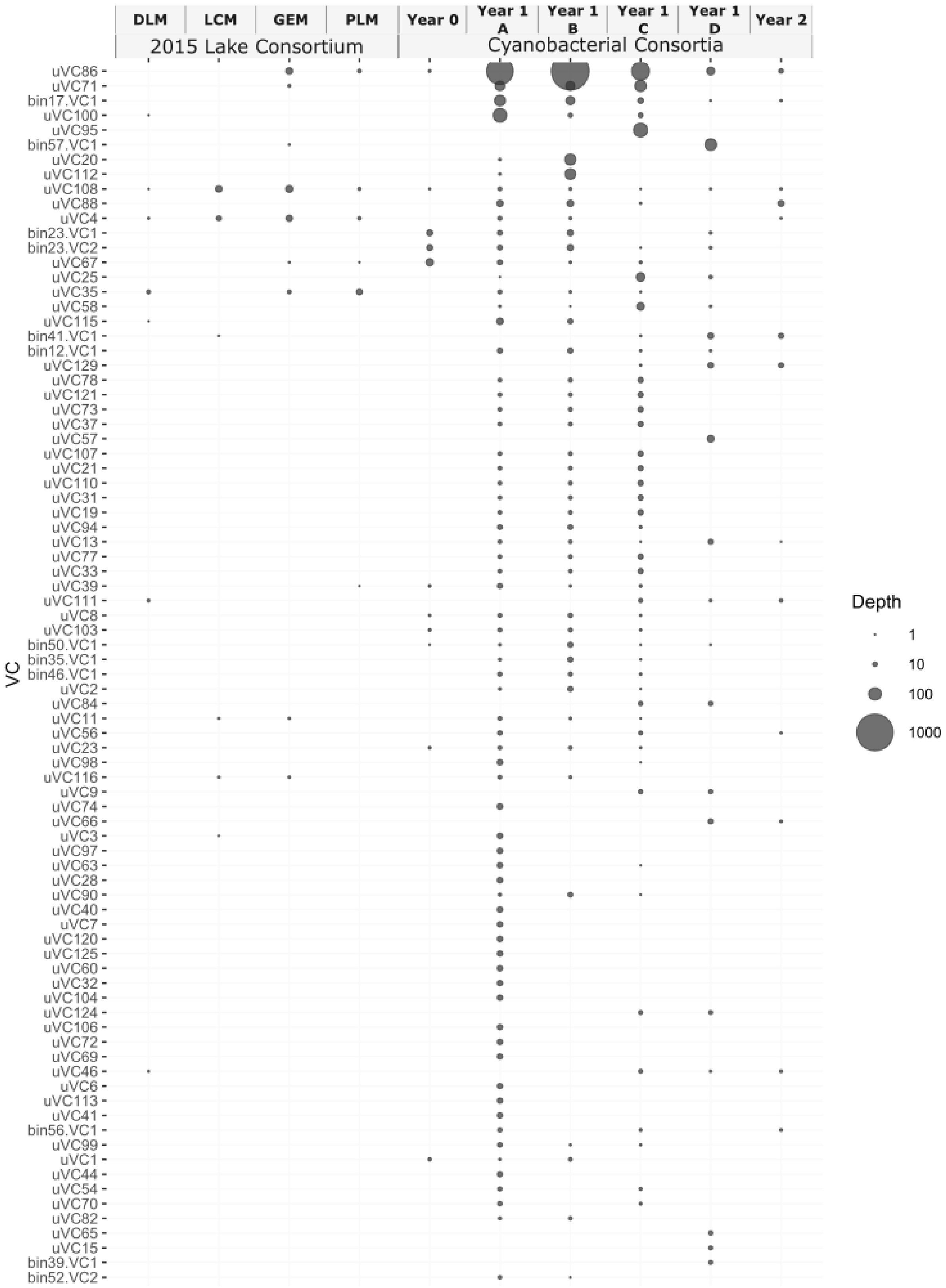

Supplementary Figure 1. A full version of Figure 2, extended to all 83 viral contigs. Viral Contig abundance. Numbers of reads mapped to all viral contigs (VCs) from all studied metagenomes. For year 1, viral contigs were found in the Year 1 metagenome from the following samples: A) Low-pH Ammonia, B) Low-pH Nitrate, C) High-pH Nitrate, D) High-pH Urea.

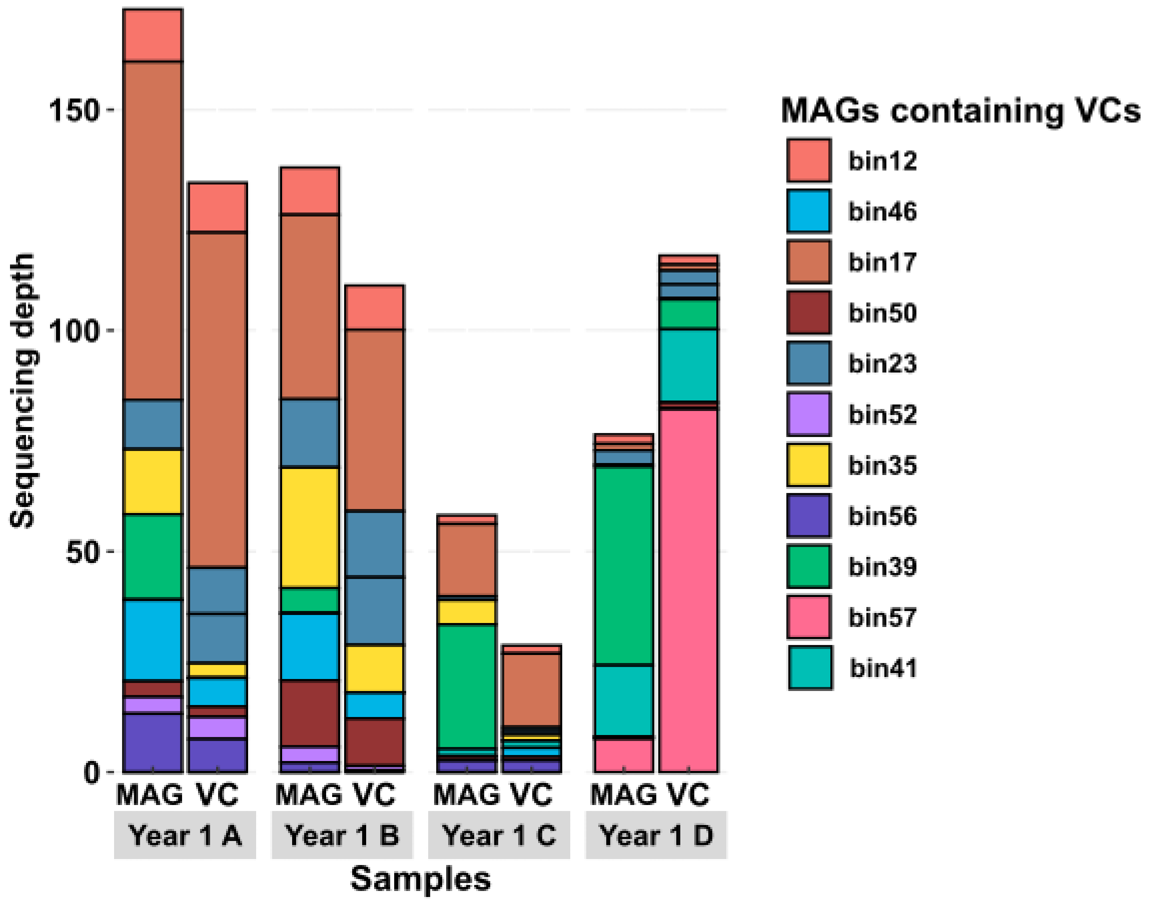

Supplementary Figure 2. Abundances of MAGs and the viral contigs (VCs) that were binned together with the MAG across four samples in the Year 1 metagenome. A) Low-pH Ammonia, B) Low-pH Nitrate, C) High-pH Nitrate, D) High-pH Urea.

Captions of Supplementary Tables

Supplementary Table 1. Metadata on all the metagenomes and their references used in this study

Supplementary Table 2. Table of Metagenome-Assembled-Genomes (MAGs) and their CRISPR-Cas system information.

Supplementary Table 3. Abundance, taxonomic and host prediction metadata of all 83 viral contigs

Supplementary Table 4. Host prediction methods and their results of viral contigs.
